# About Chediak-Higashi, Hemoglobin Lansing, and Hemoglobin Jabalpur

**DOI:** 10.4274/tjh.2014.0393

**Published:** 2015-02-15

**Authors:** Şinasi Özsoylu

**Affiliations:** 1 Retired Professor of Pediatrics, Hematology, and Hepatology, Honorary Fellow of American Academy of Pediatrics, Honorary Member of American Pediatric Society

**Keywords:** Chediak-Higashi, Hemoglobin Lansing, Hemoglobin Jabalpur

## TO THE EDITOR

I would like to express my concerns about some of the published letters in a recent issue of this journal.

About “A Rare Cause of Recurrent Oral Lesions: Chediak-Higashi Syndrome” by Karabel et al. [1], I feel that unbelievably low neutrophil counts (157/L ?) and/or coinciding presence of another hereditary disorder such as acatalasemia [2] should also be looked for as a cause of oral lesions.

It was nice seeing the addition of “Hemoglobin Lansing (Alpha) [HBA2 CD87 (HIS>GLU) (C>A)] in a Turkish Individual Resulting from Another Nucleotide Substitution” to the hemoglobinopathy library in our country by Akar et al. [3]. More interestingly, these studies were carried out in a hematologically almost normal female (hemoglobin: 13.1 g/dL; MCV: 95 fL) for premarital counseling! The authors should give an explanation for this counseling in a female in the absence of family history and the presence of normal hemoglobin level.

Dr. Çolak and her colleagues [4] also added another hemoglobin to the Turkish hemoglobinopathy library with their letter entitled “First Observation of Hemoglobin Jabalpur [Beta3 (NA3) Leu >Pro] in the Turkish Population”. Since findings of reticulocyte count, plasma hemoglobin level, peripheral smear, etc. were not given, the cause of mild anemia in the proband and his mother should be studied.
