# Supplementary material for: About Chediak-Higashi, Hemoglobin Lansing, and Hemoglobin Jabalpur
Source: Turk J Haematol. 2015 Feb 15;32(1):90–1. doi: 10.4274/tjh.2014.0393 (PMC4439918; doi:10.4274/tjh.2014.0393)
Supplement: Related texts

# Reply: Authors' Response

**Authors:** Nejat Akar

## REPLY

In Turkey, several issues including a mass screening program for the prevention of hemoglobinopathies have been taken since 2000. A hemoglobinopathy control (prevention) program was begun in 33 provinces in 2003 according to the regulations released by the Turkish Ministry of Health [1].

Our patient was subjected to hemoglobin electrophoresis prior to marriage. When abnormal hemoglobin was detected, she was sent for genetic counseling to our center.

## References

[1] Canatan D, Aydinok Y, Kılınç Y, Karakaş Z, Şaşmaz I, Apak H, Sarper N (2013). National Thalassemia Prevention Campaign: The Talotır Project. Turk J Hematol.
